# *Mycobacterium tuberculosis* Drug Resistance, Abkhazia

**DOI:** 10.3201/eid1103.040903

**Published:** 2005-03

**Authors:** Manuela Pardini, Elisabetta Iona, Francis Varaine, Hayk Karakozian, Herchanik Arzumanian, Lara Brunori, Graziella Orefici, the LONG-DRUG Study Group, Lanfranco Fattorini

**Affiliations:** * Istituto Superiore di Sanità, Rome, Italy;; † Médecins Sans Frontières, Paris, France;; ‡ Guliripchi Tuberculosis Hospital, Sukhumi, Abkhazia

**Keywords:** drug resistance, *Mycobacterium tuberculosis*, tuberculosis, Abkhazia, antituberculous agents, letter

**To the Editor:** Drug-resistant tuberculosis (TB) has been identified as a major problem in the former Soviet Union, and was recently surveyed in the Aral Sea regions of Dashoguz (Turkmenistan) and Karakalpakstan (Uzbekistan) (1). However, few data are available for the Caucasian region and published reports have focused mainly on prisons (2,3).

We report a drug resistance survey for first- and second-line anti-TB drugs conducted in Abkhazia, a Caucasian region of 8,600 km^2^ with approximately 250,000 inhabitants, at the western end of Georgia on the Black Sea. The collapse of the Soviet Union lead to disruption of TB control activities in all Eastern bloc regions (4). In Abkhazia, the shortage and poor quality of drugs, self-medication, and poor adherence to the therapy became even more evident during the war with Georgia in 1993 and the international embargo that followed. A TB program based on the World Health Organization/International Union against Tuberculosis and Lung Disease (WHO/IUATLD) recommendations was initiated in Abkhazia with the support of Médecins Sans Frontières (MSF) in 1999. In 2000, monitoring of drug resistance was started for new cases and previously treated case-patients. The study was performed in collaboration with the Guliripchi TB Hospital, MSF, and the Istituto Superiore di Sanità (ISS), a WHO/IUATLD Supranational Reference Laboratory for anti-TB drug resistance.

Sputa were collected from all patients attending Guliripchi TB Hospital in Sukhumi, the capital of Abkhazia, from September 2000 to April 2004. Patients were either referred by their practitioners or came spontaneously because TB was suspected. Diagnosis, treatment, and hospitalization were provided free. Samples were treated as previously described (5). Of 489 sputa collected from individual patients, 447 were culture positive (246 from new case-patients and 201 from previously treated case-patients) and 42 were culture negative; of these, >90% showed a negative, doubtful, or 1+ smear result. Susceptibility to first-line (streptomycin, isoniazid, rifampin, and ethambutol) and second-line (kanamycin, ethionamide, capreomycin, cycloserine, *p*-aminosalicylic acid, and ofloxacin) drugs was determined by the proportion method on Middlebrook 7H10 agar. The critical concentrations used were streptomycin, 2 µg/mL; isoniazid, 0.2 µg/mL; rifampin, 1 µg/mL; ethambutol, 5 µg/mL; kanamycin, 5 µg/mL; ethionamide, 5 µg/mL; capreomycin, 10 µg/mL; *p*-aminosalicylic acid, 2 µg/mL; and ofloxacin, 2 µg/mL (6–8). Cycloserine was used at a concentration of 30 µg/mL (9). If a strain was resistant to >1 first-line drugs, the susceptibility to all second-line drugs was determined.

Data on resistance to the first- and second-line drugs are given in the Table. The strains isolated from 35.8% of the new cases and 57.2% of the previously treated case-patients were resistant to >1 first-line drugs. The highest monoresistance was seen for isoniazid and streptomycin in both new and previously treated case-patients, while monoresistance to rifampin and ethambutol was low (<1%). Multidrug-resistant (MDR) strains (i.e., strains resistant to at least isoniazid and rifampin) were observed in 4.9% of the new cases and 25.4% of the previously treated case-patients. Strains resistant to isoniazid and streptomycin were isolated from 6.9% of the new case-patients and 8% of the previously treated case-patients. Resistance to second-line drugs was high (15.9% in new cases and 35.7% in previously treated case-patients), with the highest values being observed for kanamycin (4.5% in new cases and 21.7% in previously treated case-patients) and ethionamide (8% in new cases and 16.5% in previously treated patients). Twenty-five percent and 52.9% of the MDR strains isolated from new and previously treated case-patients, respectively, showed resistance to >1 second-line drugs.

**Table:** First-line and second line antituberculosis drug resistance in 447 *Mycobacterium tuberculosis* strains collected in Abkhazia from September 2000 to April 2004*

	No. new cases (%)	No. previously treated cases (%)	Total no. (%)
Total tested	246 (100)	201 (100)	447 (100)
Any first-line resistance	88 (35.8)	115 (57.2)	203 (45.4)
Monoresistance			
H only	31 (12.6)	28 (13.9)	59 (13.2)
S only	20 (8.1)	11 (5.5)	31 (6.9)
R only	1 (<1)	1 (<1)	2 (<1)
E only	2 (<1)	1 (<1)	3 (<1)
Any drug resistance			
Any H resistance	65 (26.4)	102 (50.7)	167 (37.4)
Any S resistance	51 (20.7)	80 (39.8)	131 (29.3)
Any R resistance	13 (5.3)	52 (25.9)	65 (14.5)
Any E resistance	14 (5.7)	35 (17.4)	49 (11)
H and R resistance			
MDR†	12 (4.9)	51 (25.4)	63 (14.1)
HRES only	7 (2.8)	24 (11.9)	31 (6.9)
HRS only	4 (1.6)	22 (10.9)	26 (5.8)
HRE only	0	3 (1.5)	3 (<1)
HR only	1 (<1)	2 (<1)	3 (<1)
H + other resistances			
HS only	17 (6.9)	16 (8)	33 (7.4)
HES only	3 (1.2)	7 (3.5)	10 (2.2)
HE only	2 (<1)	0	2 (<1)
R + other resistances (RE, RS, or RES only)	0	0	0
Total tested to second-line drugs	88 (100)	115 (100)	203 (100)
Any second-line resistance	14 (15.9)	41 (35.7)	55 (27.1)
KM	4 (4.5)	25 (21.7)	29 (14.3)
ETH	7 (8)	19 (16.5)	26 (12.8)
CM	3 (3.4)	6 (5.2)	9 (4.4)
PAS	2 (2.3)	4 (3.5)	6 (3)
OFL	0	3 (2.6)	3 (1.5)
CS	0	0	0
Total MDR strains resistant to second-line drugs‡	3 (25)	27 (52.9)	30 (47.6)
MDR + KM	2 (16.7)	10 (19.6)	12 (19)
MDR + ETH	0	5 (9.8)	5 (7.9)
MDR + KM + ETH	0	5 (9.8)	5 (7.9)
MDR + others§	1 (8.3)	7 (13.7)	8 (12.7)

*H, isoniazid; S, streptomycin; R, rifampin; E, ethambutol; KM, kanamycin; ETH, ethionamide; CM, capreomycin; PAS, *p*-aminosalicylic acid; OFL, ofloxacin; CS, cycloserine. †MDR, multidrug resistant (resistant to at least H and R). ‡Values in parenthesis are the percentages of MDR strains. §For new cases: MDR + KM + CM + PAS (1 strain); for previously treated cases: MDR + KM + CM (2 strains), MDR + KM + PAS (1 strain), MDR + KM + ETH + CM (1 strain), MDR + ETH + OFL (1 strain), MDR + PAS (1 strain), MDR + PAS + CM (1 strain).

Few data have been reported on drug resistance to first- and second-line drugs in the former Soviet Union and in the Caucasian region (1–4). Overall, in Abkhazia, monoresistance to isoniazid was higher than in Karakalpakstan and Dashoguz (1), while monoresistance to streptomycin was lower. MDR-TB in new and previously treated case-patients showed levels intermediate between these 2 regions. Resistance to kanamycin and ethionamide was 14.3% and 12.8%, respectively, while resistance to ofloxacin was low (1.5%). Fluoroquinolones have not been commonly used in Abkhazia and former regions of the Soviet Union. Currently, regimens for the treatment of MDR-TB in Abkhazia combine an intensive phase for a minimum of 6 months with at least 4 drugs to which the MTB strain is susceptible, including 1 parenteral agent and 1 fluoroquinolone (ofloxacin), followed by a continuation phase of at least 15 months with >3 drugs.

This is the first survey reporting drug susceptibility data for MTB within the Caucasus. It indicates that the prevalence of MDR strains is similar to that in other central Asia regions (1). Our results are representative of the present situation in Abkhazia since sampling systematically covered all TB cases for the period examined. The Guliripchi TB Hospital of Sukhumi is the only TB treatment center in the region, and all cases were included in the study. Overall, our data show that second-line drug resistance is present in Abkhazia, particularly among cases with MDR, and suggest the adoption of strategies for access and correct use of second-line drugs (10).
